# Flow Cytometric Detection of Waterborne Bacteria Metabolic Response to Anthropogenic Chemical Inputs to Aquatic Ecosystems

**DOI:** 10.3390/cells14050352

**Published:** 2025-02-28

**Authors:** Jill A. Jenkins, Scott V. Mize, Darren Johnson, Bonnie L. Brown

**Affiliations:** 1 U.S. Geological Survey, Wetland and Aquatic Research Center, 700 Cajundome Blvd., Lafayette, LA 70506, USA; 2 U.S. Geological Survey, Lower Mississippi-Gulf Water Science Center, Baton Rouge, LA 70816, USA; svmize@usgs.gov; 3 Cherokee Nation System Solutions, Contractor to the U.S. Geological Survey, Wetland and Aquatic Research Center, Lafayette, LA 70506, USA; johnsond@contractor.usgs.gov; 4 Department of Biological Sciences, University of New Hampshire, Durham, NH 03824, USA; bonnie.brown@unh.edu

**Keywords:** flow cytometry, biomarker, atrazine, tylosin, organic wastewater contaminants

## Abstract

Typical investigations into the biological consequences of suspected xenobiotics or nutrients introduced in watersheds include analytical chemistry screens of environmental samples—such as periphyton responses or studies of fish condition—which are all costly in terms of equipment, reagents, time, and human resources. An alternative is to assess pollutant effects on waterborne bacteria. A flow cytometric method was developed to yield rapid, same-day results that could be used to proactively screen for suspected chemical inputs into watersheds using water sampling methods that are identical to those in standard use. The analytical methods are microbe cultivation-independent, for use with waterborne bacteria that are typically viable but not culturable. The procedure is quick and inexpensive, generating measures of bacterial esterase that reflect metabolic activity and are sensitive and statistically robust. After phosphate-EDTA incubation to increase cell wall permeability, staining was performed with 5(6) carboxyfluorescein diacetate (enzyme activity) and propidium iodide (cell viability) with three bacterial species in exponential phase growth having been incubated with organic wastewater compounds (atrazine, pharmaceuticals [17α-ethynylestradiol and trenbolone], and antimicrobials [tylosin and butylparaben]). This method successfully detected metabolic changes in all bacterial species, with atrazine inducing the greatest change. Additional fluorescent stains can target specific microbial structures or functions of interest in a particular watershed. This biotechnology can inform analytical chemistry and study of biota at sites of interest and has the potential to be automated.

## 1. Introduction

Risks associated with unknown, unrecognized, or suspected chemical pollutants in the aquatic environment have been a concern for environmental scientists [1,2]. Typical investigations of the quality of streams and natural waters include sampling for nutrient enrichment [3,4] screening biota for analytical chemical concentrations [5,6,7,8], and innovative analytical chemistry methods that assess complex environmental contaminant mixtures [8,9,10]. The flux of chemicals through watersheds is derived from agricultural, industrial, and domestic uses, with complexities dependent on spatial and temporal parameters [11]. Aquatic ecosystem resource managers routinely monitor water quality [12] yet atypically investigate it in conjunction with unanticipated events such as enigmatic biota declines, with an example being freshwater mussels’ mass mortalities in the Clinch River, USA [13,14,15,16], or in remediation and conservation [17] efforts. Ecosystems are subject to daily and seasonal precipitation variations or sudden events; regions dominated by karst topography can intensify inputs of such chemicals into streams [18,19,20]. Although obvious environmental interferences such as impervious surfaces or wastewater discharge can lead to the lower quality of receiving waters [21,22], the exact sources of aquatic perturbations to biota can be elusive. However, the aquatic microbiota itself reflects water condition.

Bacterial functions and responses can be measured by metabolic activity, respiration, cell counts, and viability assays [23,24,25]. Conditions inducing viable but not culturable (VBNC) states have included low temperatures, low-nutrient oligotrophic environments, osmotic shock, oxidative stress, disinfection processes, and sunlight exposure [26,27], with the survival-stressed VBNC remaining metabolically active [28,29]. Direct microscopic counts exceed viable cell counts by several orders of magnitude, whereby the standard heterotrophic plate count characterizing microbial parameters would be erroneous [27]. The rapid enumeration of total (including VBNC) microbes from environmental samples can be accomplished using fluorescent staining and bio-adhesive slides, but the slides result in substantial mortality of certain microbe strains [30].

Flow cytometry (FCM) uses in aquatic microbiology include cell counts, size measures, nucleic acid content determination, live cell numbers, enzyme function, and assessments of community homogeneity and function in water systems, as well as for wastewater management [23,28,31,32]. Fluorescent staining coupled with FCM can be applied in bacterial monitoring, quantification, and characterization in environmental waters, wastewater, industrial bioreactors, drinking water, seawater, and freshwater, as well as in studies of antibacterial action [33,34,35,36]. Flow cytometric studies targeting aquatic microbiota provide opportunities for investigating cellular processes, thus affording a better understanding of cause-and-effect relationships [33]. At the base of the food web, microbes drive important ecosystem processes, where pollutants and their metabolites can alter community structure [37]. Given that the size, mass, nucleic acid, and protein content of bacteria are approximately 1/1000 the magnitude of those same parameters in eukaryotic cells, designing multiparametric staining protocols that work across a wide range of bacterial species can be problematic [24,38].

To explore the capacity of microbiota to reflect ambient water quality, we advanced methods using FCM for measuring bacterial metabolic activity. Because bacterial respiratory and enzymatic activity are generally higher in nutrient-rich and polluted aquatic environments [25], the hypothesis was that the influence of a swine confined animal feeding operation (CAFO) at a tributary’s confluence with a large oligotrophic river (Buffalo National River, Arkansas, AR, USA) would increase aquatic bacterial metabolic activity there, as compared to up- and down-stream sites [18]. Nitrogen and phosphorus levels in animal manure produced at CAFOs are copious, often entering watersheds through runoff, percolation into groundwater, and the connectivity of surface and groundwater systems [39]. However, in our oligotrophic river study, bacterial esterase activity was unexpectedly significantly lowest (~56%) at the CAFO-influenced site for 71% of the months (12 of 17) [18]. CAFOs also can be a source of androgens, estrogens, glucocorticoids, and antibiotics in the environment [29,40]. Thus, to investigate if unidentified anthropogenic compounds could have been influential [41], organic wastewater compounds (OWCs) previously detected in this watershed were tested on specific bacterial strains that did or could occur in the watershed [18], whereby this current presentation elaborates on flow cytometric nuance not previously described [42].

The aim of the current study is to highlight this FCM method for use with waterborne bacteria; in this case, the endpoint was metabolic activity. It does not depend on growing the bacteria on Petri plates and can be tailored to address questions relevant to the studies at hand. Monitoring for nutrients is routine for U.S. Geological Survey (USGS) investigations, yet no concurrent way to sample and study bacterial function is operative. The results of this study demonstrate the utility of measuring microbial enzyme function as a biomarker reflecting OWC presence. In conducting aquatic ecosystem conservation studies, prior to implementing resource-intensive analytical chemistry detections, Ames tests, or laboratory studies on plankton, periphyton, or fish health and reproductive condition at a site of concern for potential OWC input in vulnerable watersheds [21,43], this FCM method is a useful predictor based on evaluating physiological activity of bacteria from the water column. The field water sampling was identical to that in current use, thus serving as a complement to typical nutrient sampling [12], and it can aid in ascertaining whether there is a need for further water testing or in-depth analytical chemistry of water or biota (Table 1).

## 2. Materials and Methods

### 2.1. Processing Bacteria from River Water Samples

Water from Buffalo National River, AR, was collected at a tributary’s confluence with a large CAFO, as well as two sites up-stream and three down-stream, by using sterile replicate 250 mL bottles (*n* = 33 samplings) [12,18] sent on wet ice overnight to the laboratory for metabolic testing within 24 h. Field sampling occurred monthly on two consecutive days for 17 months from June 2017–November 2018. Once water reached 24 °C, bottles were inverted for bacterial resuspension, and then 200 mL of water was filtered through 7 μm nylon mesh (Component Supply, Sparta, TN, USA) to remove algae and large particulates. Using vacuum filtration (EMD Millipore Corporation, Billerica, MA, USA) with 0.22 μm membranes (EM Millipore Corp., Tullagreen Carrigtwohill County Cork, Ireland), filtrate was concentrated to 10 mL, then resuspended 15× for staining to measure enzyme activity, as well as for spread-plating to determine numbers of culturable colony forming units (CFUs) and fixation for cells for counts including viable, non-viable, and VBNC by FCM [18]. To ensure sterility of the filtering unit in between water samples, the 0.22 μm membrane was discarded, the unit was rinsed with 70% ethanol, and then the membrane was rinsed three times with full unit volumes of filtered, autoclaved water.

### 2.2. Bacterial Controls for Culturability and Metabolic Staining

For use as a staining control for known dead cells, with each water sample, a 10 μL aliquot of an overnight *Escherichia coli* (ATCC 35218) at 2 × 10^6^ cells mL^−1^ was heat-killed in a water bath at 75 °C for at least 30 min. The heat-killed bacteria were spread on Luria (LB) agar to ensure cells were no longer viable after 48 h of culture. Prior to FCM, cultured live and dead cell controls were pelleted at 3000× *g* for 12 min and diluted to 2 × 10^6^ cells mL^−1^ in sterile, autoclaved, and filtered water. Live cells from the field samples and live *E. coli* served as positive controls, and heat-killed *E. coli* served as negative metabolic controls for FCM and CFUs. (Two other bacterial strains for the laboratory exposures with OWCs were similarly heat-killed as staining controls; refer to Section 2.5).

### 2.3. Staining for Esterase

Replicate sterile 1.5 mL microfuge tubes with 900 μL of concentrated, resuspended filtrates were pretreated for 10 min in the dark at room temperature by the addition of 90 μL of 1 M phosphate buffer (pH 8.0) and 10 μL of 50 mM ethylenediaminetetraacetic acid (J.T. Baker, Charleston, SC, USA) to increase cell permeability for staining [28,44,45]. Bacteria were then stained with 10 μL of 5(6)carboxyfluorescein diacetate (CFDA) (stock at 5 mg CFDA mL^−1^ dimethyl-sulfoxide, Life Technologies, Eugene, OR, USA) and counter-stained with 50 μL propidium iodide (PI; Sigma-Aldrich Chemical Co., St. Louis, MO, USA) modified from [25,28]. The PI staining solution included 0.5 mg mL^−1^ PI in 0.1 M Tris-HCl and 0.1 M NaCl (pH 7.5) made fresh for use over two days. Cell-permeant CFDA nonspecific substrate measures enzymatic hydrolysis by intracellular esterases yielding fluorescent carboxyfluorescein that is retained intracellularly [25]. Cell-impermeant PI penetrates inactive or compromised membranes, intercalates into double-stranded nucleic acids, and fluoresces red under blue light excitation [25].

### 2.4. Flow Cytometry

**Figure 1:**
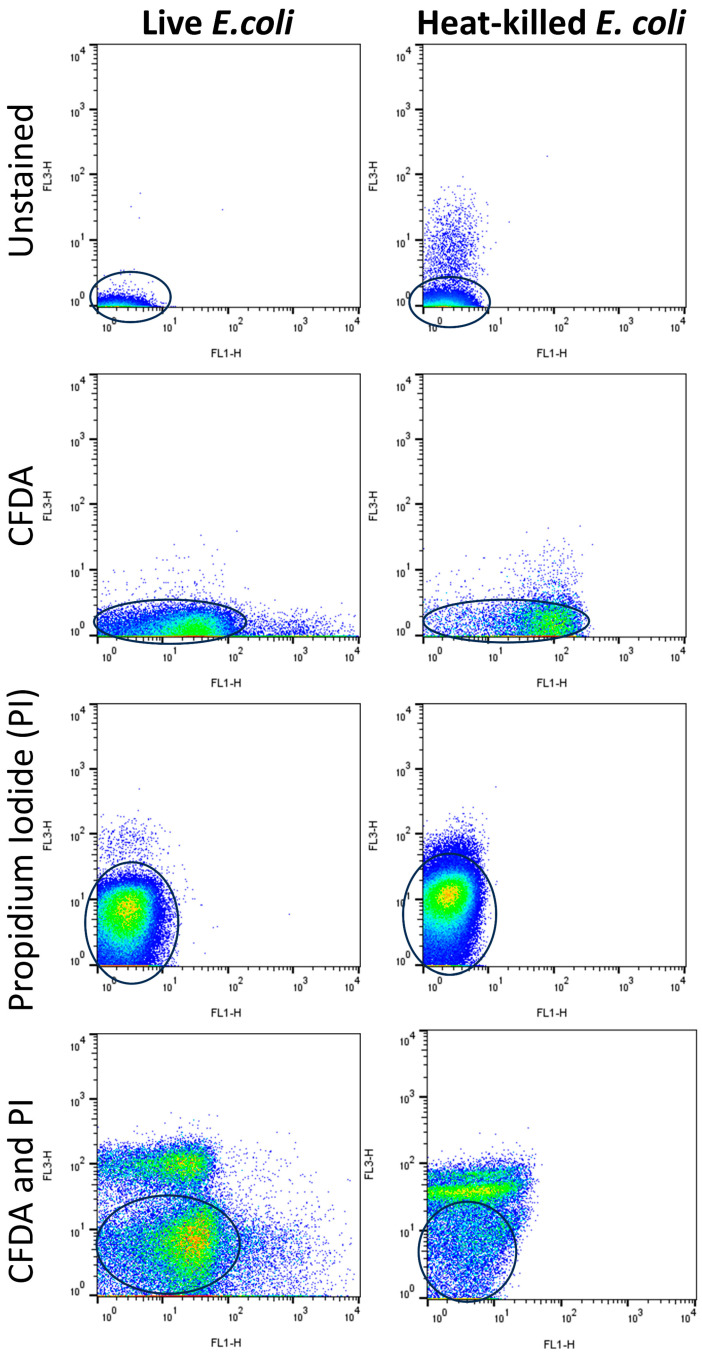
Cytograms from *Escherichia coli* (*E. coli*) staining controls for metabolic activity of live (left column) or heat-killed (right column) cells. Unstained bacteria and auto-fluorescent bacteria, concentrated near the origin, were excluded (out-gated; round gates above) for statistical computations. Bacteria stained with CDFA (5(6)-carboxyfluorescein diacetate) yield a fluorescent product because of hydrolysis by esterases (FL1-H axis; green fluorescence) shown here as populations focused at the bottom portion of the cytograms. Bacteria stained with propidium iodide (that fluoresces when intercalated into double-stranded nucleic acids of membrane-damaged cells) (FL3-H axis; red fluorescence) shown here as populations focused on the left portions of the cytograms.

Using a FACSCalibur^®^ (Becton Dickinson Immunocytometry Systems [BDIS], San Jose, CA, USA) with a 488 nm laser and CellQuestPro Version 5.2.1 software for data acquisition, data were acquired. Calibrite beads (BDIS) were used with FACSComp software v. 6.0 (BDIS) for instrument calibration. Events for each replicate were collected in duplicate at >1000 s^−1^, with ~300 K total event counts for each metabolic assay. Density plots using green (FL1-H) (CFDA) and red fluorescence (FL3-H) (PI), with FSC-H as threshold were used to present metabolic data. Unstained and auto-fluorescent cells were gated out of analyses (Figure 1). For analysis, density plots were used with FlowJo™ software, Version 10.7.0 (FlowJo Flow Cytometry Analysis Software, Ashland, OR, USA). To validate staining and optimize instrument settings, staining controls included live and heat-killed *E. coli* that were unstained, dual-stained, or individually stained with PI or 5(6)-CFDA (Figure 1).

### 2.5. Controlled Incubations with Organic Wastewater Contaminants

Based on prior findings that nutrients are higher at the CAFO site than at sites up-stream and down-stream [12] and that CFUs are highest at the CAFO while esterase activity was lowest there, the influence of potential OWCs on esterase activity was examined using bacteria that may be found in oligotrophic CAFO-associated waters [18]. Species included *E. coli* (ATCC 35218), *Streptococcus suis* (avirulent, from swine), and *S. dysgalactiae* (virulent, from silver carp, *Hypophthalmichthys molitrix*). The strains were cultured in LB broth at 35 °C until exponential phase, pelleted at 3000× *g* for 12 min, and resuspended at 2 × 10^6^ cells mL^−1^ of the selected OWC in sterile water and incubated with shaking for 2.5 and 21 h at 35 °C. The OWCs were at environmentally relevant concentrations: atrazine (herbicide), pharmaceuticals (17 α-ethynylestradiol and trenbolone), or antimicrobials (tylosin and butylparaben) (Table 2). Esterase activity was measured in triplicate at 2.5 h and 21 h, as further described [18,42,46].

### 2.6. Statistical Analyses

To measure if OWC exposure time, treatment, or concentration influenced esterase activity of bacterial species studied, activity data as proportions were arcsine(sqrt) transformed [47] and ANOVAs performed, including Tukey’s HSD multiple comparisons. Homogeneity and normality of residuals held. The alpha level was 0.05.

**Table 2:** Laboratory exposures of bacterial species with organic wastewater contaminants at low or high concentrations ^1^ in context with aquatic environmental concentrations derived from the scientific literature.

Compound	Source	Low	High	References
Atrazine	Sigma ^2^, cat. 49085 CAS 1912-24-9	3 μg L^−1^	10 μg L^−1^	[48,49,50,51,52,53,54,55]
17α-ethynylestradiol	Sigma, cat. E4876 CAS 57-63-6	5 ng L^−1^	25 ng L^−1^	[1,52,56,57]
17β-trenbolone	Sigma, cat. T3925 CAS 10161-33-8	40 ng L^−1^	80 ng L^−1^	[58,59]
Tylosin tartrate	Sigma, cat. 33847 CAS 74610-55-2	500 μg L^−1^	1000 μg L^−1^	[10,60,61,62]
Butylparaben ^3^	Spectrum ^4^, Cat. BU115 CAS 94-26-8	12.5 ng L^−1^	25 ng L^−1^	[2,63,64,65,66,67]

^1^ Flow cytometry FCS files are in [42]. ^2^ Sigma-Aldrich, Burlington, MA, USA. ^3^ Required initial dissolution in acetone. ^4^ Spectrum Chemical, New Brunswick, NJ, USA.

## 3. Results

### 3.1. Bacterial Controls

**Figure 2:**
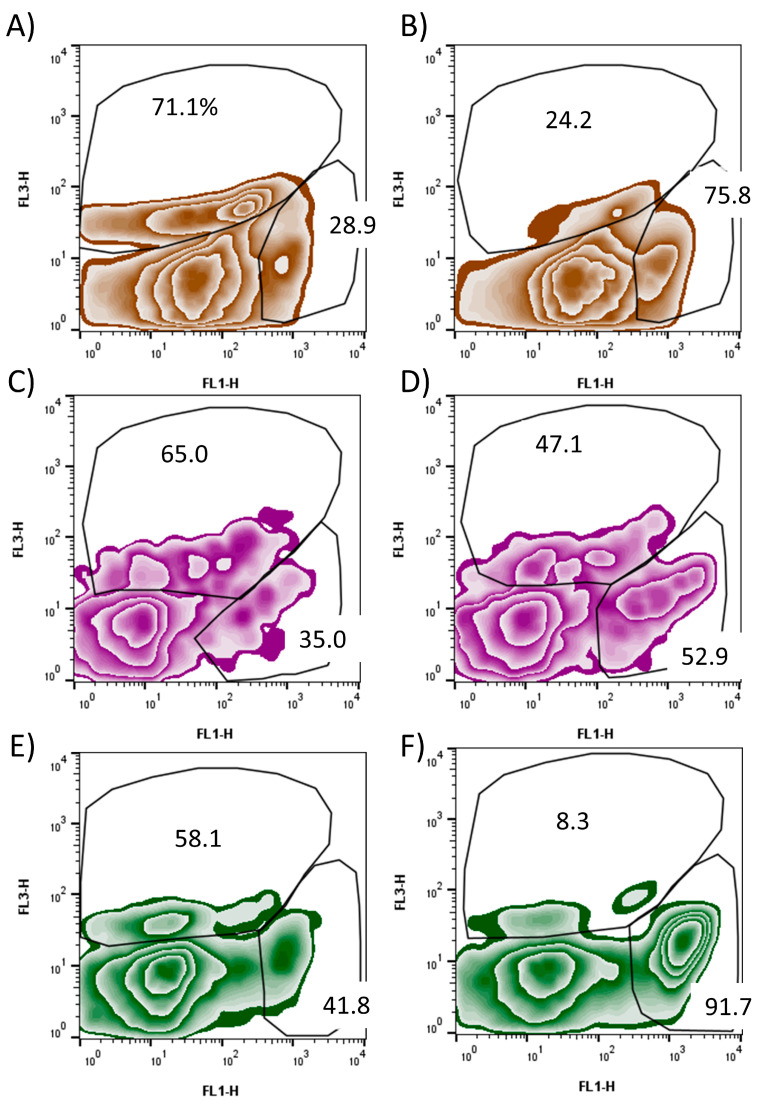
Representative flow cytometric zebra plots display representative analyses of metabolic activity in strains of bacteria that were laboratory-exposed to organic wastewater compounds. Percentages of stained cells are presented, whereby the upper gate is that of the metabolically inactive cells and the right-hand gate shows the metabolically active percentage. In panels (**A**,**B**): *Escherichia coli* was incubated with atrazine and tylosin, respectively. (**C**,**D**): *Streptococcus suis* was incubated with trenbolone and butylparaben, respectively. (**E**,**F**): *Streptococcus* dysgalactiae was incubated with atrazine and tylosin, respectively. Staining with 5(6)-carboxyfluorescein diacetate yields a fluorescent product upon hydrolysis by esterases (horizontal axis; FL1-H; green fluorescence), and propidium iodide counter-stains nucleic acids in membrane-damaged cells (vertical axis; FL3-H; red fluorescence). Unstained or auto-fluorescent particles were out-gated for the analyses (e.g., ungated population near the origin) (Figure 1).

Live and heat-killed *E. coli* suspensions in equal volumes were analyzed using FCM contour and density plots performed as expected in the bi-color fluorescence assay for esterase, with density plots employed for data analyses [18]. Only clearly stained cells were enumerated for the analysis gating of metabolic and non-metabolic cells (Figure 2), whereby unstained and auto-fluorescent populations and debris were out-gated (Figure 1).

### 3.2. Bacterial Exposures to Contaminants

The OWCs that previously were detected in an oligotrophic watershed [18] influenced esterase activity in this study. Overall, the bacterial species were differentially susceptible to compound exposure, with metabolic inhibition levels varying with exposure time and compound, thus substantiating that xenobiotics entering waterways are influencing microbial ecology. Atrazine decreased metabolic activity the most of any of the xenobiotics. Esterase activity declined with exposure time and varied with OWC type but not with concentration. At 2.5 h exposure, metabolic activity was similarly inhibited among all bacterial species tested in vitro across all OWCs (Table 3); differences among species emerged at the 21 h treatment duration, and it was decreased most in *S. suis* for all xenobiotics (Table 3; Figure 2). Data are in [42,46].

## 4. Discussion

The FCM approach described here showed altered microbial enzyme activity along the stretch of an oligotrophic river alongside a CAFO [18]. The reduction in bacterial esterase activity in response to OWC exposure is proof of the concept that xenobiotic micropollutants in an aquatic ecosystem have effects at the base of the food web and alter the local microbial ecology.

In swine CAFO management, antibiotics are applied at each animal life stage for growth enhancement or treating infection, and typically, swine liquid manure is applied to farmland as fertilizer [68,69]. In this study, the macrolide antibiotic tylosin at the concentrations and times of exposure did not demonstrate the statistically significant inhibition of reductase in *S. suis* or the other bacterial strains tested (Table 3). The most likely macrolide present in agricultural runoff is tylosin [60], which is often used in swine production facilities, may not be fully metabolized in livestock, and can be excreted as the parent compound or as a metabolite [10]. Distributions of tylosin, nutrients, and agriculturally applied chemicals such as atrazine to watersheds are often episodic due to hydrological conditions and seasons [10,12,70].

Atrazine, a highly soluble herbicide, is the second most used pesticide in the United States [51] and has been among the most frequently detected compounds in agricultural streams [71]. Because of its distribution and potential environmental persistence, atrazine contamination of freshwater ecosystems will remain a concern [51]. The results of this study showed that atrazine resulted in the most decreased esterase activity compared to the other four OWCs tested (Table 3). Studies on atrazine and microbes often address compound breakdown by bacteria for soil remediation or impacts on photosynthetic processes in bacterioplankton and phytoplankton communities [72,73]. Atrazine did not directly affect the survival of fecal indicator bacteria, but it altered phytoplankton and periphyton communities, thus indirectly influencing the distribution and abundance of *E. coli* [74]. Our study is the first to show that atrazine directly affected metabolic function in bacteria (Table 3). Moreover, atrazine reduces reproduction in fathead minnows (*Pimephales promelas*) [75], thus underscoring the potential utility of this flow cytometric method for use as an early warning assessment for aquatic ecosystems of interest.

As part of metabolic activity, cells operate a suite of esterases and dehydrogenases that are linked to the respiratory activity of metabolically active cells [76]. Esterases represent a diverse group of hydrolases distributed in animals, plants, and microorganisms that catalyze the cleavage and formation of ester bonds [77]. Being stable and not requiring cofactors, they are involved in microbial catabolism and peptidoglycan maintenance [77,78]. Dehydrogenases are oxidoreductases occurring intracellularly in all microbial cells and are directly linked with respiratory processes [76,79]. Another enzyme of potential interest is superoxide dismutase, which responds to reactive oxygen species (ROS) generated during oxidative stress. An increase in ROS may be detected using dihydroethidium (HE), where the oxidation of HE results in the cleavage of ethidium which fluoresces with the intercalation with DNA [80]. Comparing the standard direct plate count with rapid FCM enzyme measurements, metabolic activity is reflected by FCM despite stressed cells likely having entered a non-culturable state [76,81]. Various nucleic acid stains can be added, with attention being paid to the concentrations, potential permeabilization methods, incubation temperatures and times [80].

Butylparaben — a preservative in foodstuffs, cosmetics, and pharmaceuticals — as well as the hormones used in this study, act as endocrine disrupter chemicals [66,82,83]. The results showed that trenbolone’s effects on esterase activity in *S. suis* were increased with time compared to butylparaben (Table 3). Trenbolone is more influential with time in *S. suis*, but at 2.5 h EE2 was more inhibitive than trenbolone (Table 3). Further direct bacterial exposures with single compounds would be insightful. In *S. dysgalactiae*, time and treatment interactions showed that atrazine was significantly inhibitive (Table 3).

Freshwater microbial communities exhibit high compositional and functional variability across spatial and temporal scales, reflecting changes in water chemistry and hydrodynamic processes occurring in time and space [11,84,85]. The extents of impervious surfaces, urbanization, agriculture, and nutrients vary greatly across large ecoregions and smaller landscapes, with all of them influencing watersheds and interconnected biota and microbiota [11,21,84,86]. Contaminants of emerging concern such as OWCs enter water systems from non-point sources, introducing fecal matter, runoff, and landfill leeching, and from point sources such as combined sewer overflows, failed septic systems, spills, and industrial outflows. Many of these contribute to multiple pollutant types such as pesticides, industrial compounds, nutrients, and hormones [82]. These OWCs can persist in freshwater and are typically present in low environmental concentrations (ng/L to μg/L range), yet they vary among water compartments such as influent, effluent, surface or groundwater, or estuaries (Table 2) [2,8,62,82]. All aquatic organisms are exposed to OWCs in a contaminated system; in eukaryotes, the consequences of pollutants triggering sublethal toxicity on physiological pathways are underestimated [2]. Some studies on reproductive effects in male fish have demonstrated organismal and cellular effects [6,7,43,87,88]. Prioritizing the detection and risk assessment of OWC pollution is difficult because sub-lethal concentrations vary, mixtures are pervasive, and consequent physiological effects at sub-lethal concentrations are not clearly established per compound [1,2,8].

Because the primary microbial entities and communities are vulnerable to pollutants [62], the FCM detection of heterotrophic functional changes in natural microbial communities in aquatic ecosystems can offer an early warning for further studies at higher levels of biological organization to investigate linkages among potential anthropogenic inputs and organismal responses, as well as other applications (Table 1). With its notable sensitivity, a FCM approach comparing aquatic microbial function from reference and potentially contaminated sites could serve as a prescreening option prior to more extensive studies on animal conditions and costly analytical chemistry detections.

Although FCM has become an increasingly applied tool in aquatic microbiology [89], the smaller size and general cellular characteristics of bacteria can present challenges in protocol development as compared with eukaryotic cells [38]. Bacteria behave differently in the interaction with reagents, whereby the cell wall and the presence of efflux pumps and pores may differ from eukaryotes, and Gram-negative bacteria exclude lipophilic or hydrophilic molecules. Chemicals such as citrate or ethylenediaminetetraacetic acid (EDTA), as used in this experiment, may be used to increase cell wall permeability [28,38,45,80,90]. Gram-positive nucleic acid staining has been variable among species [91], with staining combinations shown to be effective in assessing Gram and viability status in complex mixtures [92].

Analysis at the single cell level is required for insight into cellular responses to stresses, whereby structural variations, membrane and wall charges, intracellular contents, metabolites, species and subpopulation identifications within consortia, as well as enzyme activity have been measured in freshwater and marine studies and in medical device sterilization studies [23,76,77]. Generally, dyes used to monitor enzymatic activity are cleaved upon uptake, leading to the production of a fluorescent signal [38]. As with this study, carboxyfluorescein diacetate (cFDA) is cleaved by esterases to release the fluorescent fluorescein; pH conditions may affect fluorescein’s fluorescence emission intensity, with pH 9 being the most intense [80]. As novel stains and markers continue to be developed for use with flow cytometry, more microbial biomarker responses can be measured [93]. One advantage of this FCM is the immediate measurement of functions of microbiota in the watershed; one disadvantage is the skill level needed to perform FCM.

Flow cytometric analyses for measuring specific aquatic bacterial parameters of interest from ecosystems of concern can be tailored to hypotheses ranging in topical areas from listed species in riverine systems to questionable outfalls above or below drinking water sources. The sampling of the water was performed using USGS guidelines in conjunction with routine nutrient monitoring [12]. These data provide proof of concept that individual OWCs influence the metabolic function of primary producers in an ecosystem and may provide impetus for further studies.

Summarily, to explore the capacity of microbiota to reflect water quality, we advanced methods using FCM for measuring bacterial metabolic activity. After exposure to three bacterial species, atrazine decreased bacterial esterase over tylosin, butylparaben, EE2, and trenbolone. This method can serve to prescreen aquatic conditions prior to more costly investigations into aquatic animal conditions or analytical chemistry. Microbial primary producers are vulnerable to pollutants.

## Figures and Tables

**Table 1:** Examples of applications for analysis by flow cytometry of metabolic activity of heterotrophic bacteria from aquatic ecosystems. OWC = organic wastewater compounds.

Adjunct to Routine Nutrient Monitoring
Adjunct to routine fecal indicator bacteria testing
Analysis of drinking water for safe management practices
Up- and down-stream investigations of suspected OWC inputs to explain ecosystem service change (e.g., mussel habitat loss)
Monitor microbial function along with gene expression and metabolic profiles Monitoring wastewater treatment plant operations Complement to investigations of specific antibiotic resistance elements and minimum inhibitory concentrations Nonpoint source management, Section 319 of the Clean Water Act (CWA§319) Complement to community structure analysis ^1^

^1^ Refer to [18].

**Table 3:** Statistical main effects and two-way interactions of inhibition of reductase activity measured by flow cytometry after exponential phase bacterial strains were exposed to organic wastewater compounds (OWCs) ^1^ that had been, or were likely to occur, in Buffalo National River ^2^.

Bacteria	OWC	Concentration ^3^	Incubation Time (h)	Interaction OWC and Time	Signif. Pairs for Two-Way Time and Treatment Interaction
*Escherichia coli*	Az > Ty = Bp = EE_2_ = Tb		2.5 > 21		
	*p* < 0.0001	ns	*p* < 0.0001	ns	
*Streptococcus suis*	ns	ns	ns	*p* = 0.0001	Within 2.5 h Bp > Tb (*p* < 0.0015)
					EE_2_ > Tb (*p* < 0.0001)
					Within 21 h Tb > Bp (*p* = 0.0010)
					Within Tb 21 > 2.5 (*p* = 0.0004)
*Streptococcus dysgalactiae*	Az > Tb = Ty = Bp = EE_2_	ns	2.5 > 21	*p* = 0.0024	Within 2.5 h Az > Bp (*p* = 0.0010)
	*p* < 0.0001		*p* < 0.0001		Az > EE_2_ (*p* < 0.0010)
					Ty > EE_2_ (*p* = 0.0013)
					Within 21 h Az > Bp (*p* < 0.0001)
					Az > EE_2_ (*p* = 0.0006)
					Az > Tb (*p* < 0.0001)
					Az > Ty (*p* < 0.0001)
					Within Tb 2.5 > 21 (*p* < 0.0001)

^1^ Az = atrazine at 3 and 10 ng L^−1^; Bp = butylparaben at 12.5 and 25 ng L^−1^; EE_2_ = α–ethynylestradiol at 5 and 25 ng L^−1^; Tb = trenbolone 40 and 80 ng L^−1^; Ty = tylosin 500 and 1000 µL^−1^. ^2^ [18]; briefly each strain was incubated with the OWC at two concentrations for two time periods, and triplicate samples were analyzed. ^3^ See Table 2.

## Data Availability

Data are available at ScienceBase in two data releases: [42,46].

## References

[1] Daughton C.G., Ternes T.A. (1999). Pharmaceuticals and Personal Care Products in the Environment: Agents of Subtle Change?. Environ. Health Perspect..

[2] Archer E., Petrie B., Kasprzyk-Hordern B., Wolfaardt G.M. (2017). The Fate of Pharmaceuticals and Personal Care Products (PPCPs), Endocrine Disrupting Contaminants (EDCs), Metabolites and Illicit Drugs in a Wwrw and Environmental Waters. Chemosphere.

[3] Fuhrer G.J., Tanner D.Q., Morace J.L., McKenzie S.W., Skach K.A. (1996). Water Quality of the Lower Columbia River Basin: Analysis of Current and Historical Water-Quality Data through 1994. U.S. Geological Survey Water-Resources Investigations Report 95-4294.

[4] Justus B.G., Driver L.J., Green J.J., Wentz N.J. (2019). Relations of Dissolved-Oxygen Variability, Selected Field Constituents, and Metabolism Estimates to Land Use and Nutrients in High-Gradient Boston Mountain Streams, Arkansas. Environ. Monit. Assess..

[5] Schmitt C.J. (2002). Biomonitoring of Environmental Status and Trends (Best) Program: Environmental Contaminants and Their Effects on Fish in the Mississippi River Basin. Biological Science Report.

[6] Jenkins J.A., Olivier H.M., Draugelis-Dale R.O., Eilts B.E., Torres L., Patiño R., Nilsen E., Goodbred S.L. (2014). Assessing Reproductive and Endocrine Parameters in Male Largescale Suckers (*Catostomus Macrocheilus*) Along a Contaminant Gradient in the Lower Columbia River, USA. Sci. Total Environ..

[7] Patino R., VanLandeghem M.M., Goodbred S.L., Orsak E., Jenkins J.A., Echols K.R., Rosen M.R., Torres L. (2015). Novel Associations between Contaminant Body Burdens and Biomarkers of Reproductive Condition in Male Common Carp Along Multiple Gradients of Contaminant Exposure in Lake Mead National Recreation Area, USA. Gen. Comp. Endocrinol..

[8] Barber L.B., Keefe S.H., Antweiler R.C., Taylor H.E., Wass R.D. (2006). Accumulation of Contaminants in Fish from Wastewater Treatment Wetlands. Environ. Sci. Technol..

[9] Alvarez D.A., Petty J.D., Huckins J.N., Jones-Lepp T.L., Getting D.T., Goddard J.P., Manahan S.E. (2004). Development of a Passive, in situ, Integrative Sampler for Hydrophilic Organic Contaminants in Aquatic Environments. Environ. Toxicol. Chem..

[10] Washington M.T., Moorman T.B., Soupir M.L., Shelley M., Morrow A.J. (2018). Monitoring Tylosin and Sulfamethazine in a Tile-Drained Agricultural Watershed Using Polar Organic Chemical Integrative Sampler (POCIS). Sci. Total Environ..

[11] Barber L.B., Murphy S.F., Verplanck P.L., Sandstrom M.W., Taylor H.E., Furlong E.T. (2006). Chemical Loading into Surface Water Along a Hydrological, Biogeochemical, and Land Use Gradient: A Holistic Watershed Approach. Environ. Sci. Technol..

[12] Justus B.G., Driver L.J., Burge D.R.L. (2021). Seasonal Periphyton Response to Low-Level Nutrient Exposure in a Least Disturbed Mountain Stream, the Buffalo River, Arkansas. Ecol. Indic..

[13] Cope W.G., Bergeron C.M., Archambault J.M., Jones J.W., Beaty B., Lazaro P.R., Shea D., Callihan J.L., Rogers J.J. (2021). Understanding the Influence of Multiple Pollutant Stressors on the Decline of Freshwater Mussels in a Biodiversity Hotspot. Sci. Total Environ..

[14] Haag W.R. (2019). Reassessing Enigmatic Mussel Declines in the United States. Freshw. Mollusk Biol. Conserv..

[15] Putnam J.G., Steiner J.N., Richard J.C., Leis E., Goldberg T.L., Dunn C.D., Agbalog R., Knowles S., Waller D.L. (2023). Mussel Mass Mortality in the Clinch River, USA: Metabolomics Detects Affected Pathways and Biomarkers of Stress. Conserv. Physiol..

[16] Richard J.C., Leis E., Dunn C.D., Agbalog R., Waller D., Knowles S., Putnam J., Goldberg T.L. (2020). Mass Mortality in Freshwater Mussels (*Actinonaias pectorosa*) in the Clinch River, USA, Linked to a Novel Densovirus. Sci. Rep..

[17] Newton T.J., Cope W.G., Farris J.L., Van Hassel J.H. (2006). Biomarker Responses of Unionid Mussels to Environmental Contaminants. Freshwater Bivalve Ecotoxicology.

[18] Jenkins J.A., Draugelis-Dale R.O., Hoffpauir N.M., Baudoin B.A., Matkin C., Driver L., Hodges S., Brown B.L. (2024). Flow Cytometric Assessments of Metabolic Activity in Bacterial Assemblages Provide Insight into Ecosystem Condition Along the Buffalo National River, Arkansas. Sci. Total Environ..

[19] Kosic K., Bitting C.L., Van Brahana J., Bitting C.J. (2015). Proposals for Integrating Karst Aquifer Evaluation Methodologies into National Environmental Legislations: Case Study of a Concentrated Animal Feeding Operation in Big Creek Basin and Buffalo National River Watershed, Arkansas, USA. Sustain. Water Resour. Manag..

[20] Stadler H., Klock E., Skritek P., Mach R.L., Zerobin W., Farnleitner A.H. (2010). The Spectral Absorption Coefficient at 254 nm as a Real-Time Early Warning Proxy for Detecting Faecal Pollution Events at Alpine Karst Water Resources. Water Sci. Technol..

[21] Blazer V.S., Pinkney A.E., Jenkins J.A., Iwanowicz L.R., Minkkinen S., Draugelis-Dale R.O., Uphoff J.H. (2013). Reproductive Health of Yellow Perch *Perca flavescens* in Selected Tributaries of the Chesapeake Bay. Sci. Total Environ..

[22] Jenkins J.A., Hartley S.B., Carter J., Johnson D.J., Alford J.B. (2013). A Geographic Information System Tool for Aquatic Resource Conservation in the Red and Sabine River Watersheds of the Southeast United States. River Res. Appl..

[23] Muller S., Nebe-von-Caron G. (2010). Functional Single-Cell Analyses: Flow Cytometry and Cell Sorting of Microbial Populations and Communities. FEMS Microbiol. Rev..

[24] Sträuber H., Müller S. (2010). Viability States of Bacteria—Specific Mechanisms of Selected Probes. Cytom. Part A.

[25] Yamaguchi N., Nasu M. (1997). Flow Cytometric Analysis of Bacterial Respiratory and Enzymatic Activity in the Natural Aquatic Environment. J. Appl. Microbiol..

[26] Morishige Y., Fujimori K., Amano F. (2015). Use of Flow Cytometry for Quantitative Analysis of Metabolism of Viable but Non-Culturable (VBNC) Salmonella. Biol. Pharm. Bull..

[27] Ye C., Chen C., Feng M., Ou R., Yu X. (2024). Emerging Contaminants in the Water Environment: Disinfection-Induced Viable but Non-Culturable Waterborne Pathogens. J. Hazard. Mater..

[28] Hoefel D., Grooby W.L., Monis P.T., Andrews S., Saint C.P. (2003). A Comparative Study of Carboxyfluorescein Diacetate and Carboxyfluorescein Diacetate Succinimidyl Ester as Indicators of Bacterial Activity. J. Microbiol. Methods.

[29] Martinez J.L. (2008). Antibiotics and Antibiotic Resistance Genes in Natural Environments. Science.

[30] Franklin A.B., Campbell A.H., Higgins C.B., Barker M.K., Brown B.L. (2011). Enumerating Bacterial Cells on Bioadhesive Coated Slides. J. Microbiol. Methods.

[31] Coggins L.X., Larma I., Hinchliffe A., Props R., Ghadouani A. (2020). Flow Cytometry for Rapid Characterisation of Microbial Community Dynamics in Waste Stabilisation Ponds. Water Res..

[32] Safford H.R., Bischel H.N. (2019). Flow Cytometry Applications in Water Treatment, Distribution, and Reuse: A Review. Water Res..

[33] Besmer M.D., Weissbrodt D.G., Kratochvil B.E., Sigrist J.A., Weyland M.S., Hammes F. (2014). The Feasibility of Automated Online Flow Cytometry for in-situ Monitoring of Microbial Dynamics in Aquatic Ecosystems. Front. Microbiol..

[34] Bouix M., Ghorbal S., Picque D., Perret B., Saulou-Bérion C. (2022). A Rapid Method for the Assessment of the Vitality of Microorganisms Using Flow Cytometry. Cytom. Part A.

[35] Nieto-Velázquez N.G., Gomez-Valdez A.A., González-Ávila M., Sánchez-Navarrete J., Toscano-Garibay J.D., Ruiz-Pérez N.J. (2021). Preliminary Study on Citrus Oils Antibacterial Activity Measured by Flow Cytometry: A Step-by-Step Development. Antibiotics.

[36] Williams T.C., Woznow T., Velapatino B., Asselin E., Nakhaie D., Bryce E.A., Charles M. (2023). In vitro Comparison of Methods for Sampling Copper-Based Antimicrobial Surfaces. Microbiol. Spectr..

[37] Allison S.D., Martiny J.B.J. (2008). Resistance, Resilience, and Redundancy in Microbial Communities. Proc. Natl. Acad. Sci. USA.

[38] Shapiro H.M., Nebe-von-Caron G., Hawley T.S., Hawley R.G. (2004). Multiparameter Flow Cytometry of Bacteria. Flow Cytometry Protocols.

[39] Buckerfield S.J., Waldron S., Quilliam R.S., Naylor L.A., Li S., Oliver D.M. (2019). How Can We Improve Understanding of Faecal Indicator Dynamics in Karst Systems under Changing Climatic, Population, and Land Use Stressors?—Research Opportunities in SW China. Sci. Total Environ..

[40] Liu S., Ying G.-G., Zhou L.-J., Zhang R.-Q., Chen Z.-F., Lai H.-J. (2012). Steroids in a Typical Swine Farm and Their Release into the Environment. Water Res..

[41] Mott D.N. (2016). Permitted Concentrated Animal Feeding Operation Assessment Buffalo National River.

[42] Wood K., Boudreaux H., Jenkins J.A. (2025). Esterase Activity from Three Selected Bacterial Species Exposed to Five Organic Wastewater Compounds. U.S. Geological Survey Data Release.

[43] Goodbred S.L., Patiño R., Alvarez D.A., Johnson D., Hannoun D., Echols K.R., Jenkins J.A. (2024). Fish Health Altered by Contaminants and Low Water Temperatures Compounded by Prolonged Regional Drought in the Lower Colorado River Basin, USA. Toxics.

[44] Chrzanowski T.H., Crotty R.D., Hubbard J.G., Welch R.P. (1984). Applicability of the Fluorescein Diacetate Method of Detecting Active Bacteria in Freshwater. Microb. Ecol..

[45] Diaper J.P., Edwards C. (1994). The Use of Fluorogenic Esters to Detect Viable Bacteria by Flow Cytometry. J. Appl. Microbiol..

[46] Hoffpauir N.M., Baudoin B.A., Jenkins J.A. (2022). Characteristics of Bacteria and Water Quality Along the Buffalo National River, 2017–2018. U.S. Geological Survey Data Release.

[47] Zar J.H. (2010). Biostatistical Analysis.

[48] Demcheck D.K., Swarzenski C.M. (2003). Atrazine in Southern Louisiana Streams, 1998–2000.

[49] Bradley P.M., Journey C.A., Romanok K.M., Barber L.B., Buxton H.T., Foreman W.T., Furlong E.T., Glassmeyer S.T., Hladik M.L., Iwanowicz L.R. (2017). Expanded Target-Chemical Analysis Reveals Extensive Mixed-Organic-Contaminant Exposure in U.S. Streams. Environ. Sci. Technol..

[50] Chen N., Valdes D., Marlin C., Ribstein P., Alliot F., Aubry E., Blanchoud H. (2019). Transfer and Degradation of the Common Pesticide Atrazine through the Unsaturated Zone of the Chalk Aquifer (Northern France). Environ. Pollut..

[51] Beaulieu M., Cabana H., Taranu Z., Huot Y. (2020). Predicting Atrazine Concentrations in Waterbodies across the Contiguous United States: The Importance of Land Use, Hydrology, and Water Physicochemistry. Limnol. Oceanogr..

[52] Matkin C.Y. (2021). Cellular, Organ, and Organismal Level Effects after Exposure to a Simulated Cafo Runoff Comprising 17α-Ethynylestradiol, 17β- Trenbolone, and Atrazine in Western Mosquitofish (*Gambusia affinis*). Doctoral Dissertation.

[53] Li S., Huang F., Piao H., Li W., Liu F., Zhu Q., He Y., Wang J., Yan M. (2024). Occurrence and Distribution of Atrazine in Groundwater from Agricultural Areas in China. Sci. Total Environ..

[54] Nuchan P., Kovitvadhi U., Sangsawang A., Kovitvadhi S., Klaimala P., Srakaew N. (2025). Potential Utilization of Bivalve Hemolymph as a Biomonitoring Tool for Assessment of Atrazine Contamination. J. Hazard. Mater..

[55] U.S. Geological Survey USGS Water Data for the Nation: U.S. Geological Survey National Water Information System Database. https://waterdata.usgs.gov/nwis.

[56] Zhao L., Wang C., Sun F., Liao H., Chang H.R., Jia X. (2024). Assessment of Occurrence, Partitioning and Ecological Risk for 144 Steroid Hormones in Taihu Lake Using UPLC-MS/MS with Machine Learning Model. Chemosphere.

[57] Tang Z., Liu Z., Wang H., Dang Z.C., Liu Y. (2021). A Review of 17α-Ethynylestradiol (EE2) in Surface Water across 32 Countries: Sources, Concentrations, and Potential Estrogenic Effects. J. Environ. Manag..

[58] Morthorst J.E., Holbech H., Bjerregaard P. (2010). Trenbolone Causes Irreversible Masculinization of Zebrafish at Environmentally Relevant Concentrations. Aquat. Toxicol..

[59] Durhan E.J., Lambright C.S., Makynen E.A., Lazorchak J., Hartig P.C., Wilson V.S., Gray L.E., Ankley G.T. (2006). Identification of Metabolites of Trenbolone Acetate in Androgenic Runoff from a Beef Feedlot. Environ. Health Perspect..

[60] Huang C., Renew J.E., Smeby K.L., Pinkston K., Sedlak D.L. (2011). Assessment of Potential Antibiotic Contaminants in Water and Preliminary Occurrence Analysis. J. Contemp. Water Res. Educ..

[61] Luo Y., Xu L., Rysz M., Wang Y., Zhang H., Alvarez P.J.J. (2011). Occrrence and Transport of Tetracycline, Sulfonamide, Quinolone, and Macrolide Antibiotics in the Haihe River Basin, China. Environ. Sci. Technol..

[62] Carvalho I.T., Santos L. (2016). Antibiotics in the Aquatic Environments: A Review of the European Scenario. Environ. Int..

[63] Brausch J.M., Rand G.M. (2011). A Review of Personal Care Products in the Aquatic Environment: Environmental Concentrations and Toxicity. Chemosphere.

[64] Yamamoto H., Tamura I., Hirata Y., Kato J., Kagota K., Katsuki S., Yamamoto A., Kagami Y., Tatarazako N. (2011). Aquatic Toxicity and Ecological Risk Assessment of Seven Parabens: Individual and Additive Approach. Sci. Total Environ..

[65] Bledzka D., Gromadzinska J., Wasowicz W. (2014). Parabens. From Environmental Studies to Human Health. Environ. Int..

[66] Haman C., Dauchy X., Rosin C., Munoz J.-F. (2015). Occurrence, Fate and Behavior of Parabens in Aquatic Environments: A Review. Water Res..

[67] Song C., Lin J., Huang X., Wu Y., Liu J., Wu C. (2016). Effect of Butyl Paraben on the Development and Microbial Composition of Periphyton. Ecotoxicology.

[68] Brooks J.P., Adeli A., McLaughlin M.R. (2014). Microbial Ecology, Bacterial Pathogens, and Antibiotic Resistant Genes in Swine Manure Wastewater as Influenced by Three Swine Management Systems. Water Res..

[69] Mott D.N., Laurans J. (2004). Water Resources Management Plan: Buffalo National River Arkansas.

[70] Lerch R.N., Sadler E.J., Sudduth K.A., Baffaut C., Kitchen N.R. (2011). Herbicide Transport in Goodwater Creek Experimental Watershed: I. Long-Term Research on Atrazine. J. Am. Water Resour. Assoc..

[71] Demcheck D.K., Tollett R.W., Mize S.V., Skrobialowski S.C., Fendick R.B., Swarzenski C.M., Porter S. (2004). Water Quality in the Acadian-Pontchartrain Drainages, Louisiana and Mississippi, 1999–2001.

[72] Farkas D., Proctor K., Kim B., Rossa C.A., Kasprzyk-Hordern B., Di Lorenzo M. (2024). Assessing the Impact of Soil Microbial Fuel Cells on Atrazine Removal in Soil. J. Hazard. Mater..

[73] Yang L., Mou S., Li H., Zhang Z., Jiao N., Zhang Y. (2021). Terrestrial Input of Herbicides Has Significant Impacts on Phytoplankton and Bacterioplankton Communities in Coastal Waters. Limnol. Oceanogr..

[74] Staley Z.R., Rohr J.R., Harwood V.J. (2011). Test of Direct and Indirect Effects of Agrochemicals on the Survival of Fecal Indicator Bacteria. Appl. Environ. Microbiol..

[75] Tillitt D.E. (2010). Atrazine Reduces Reproduction in Fathead Minnow (*Pimephales promelas*). Aquat. Toxicol..

[76] McEvoy B., Lynch M., Rowan N.J. (2020). Opportunities for the Application of Real-Time Bacterial Cell Analysis Using Flow Cytometry for the Advancement of Sterilization Microbiology. J. Appl. Microbiol..

[77] Bornscheuer U.T. (2002). Microbial Carboxyl Esterases: Classification, Properties and Application in Biocatalysis. FEMS Microbiol. Rev..

[78] Weadge J.T., Pfeffer J.M., Clarke A.J. (2005). Identification of a New Family of Enzymes with Potential *O*-Acetylpeptidoglycan Esterase Activity in Both Gram-Positive and Gram-Negative Bacteria. BMC Microbiol..

[79] Beyer L., Wachendorf C., Elsner D.C., Knabe R. (1993). Suitability of Dehydrogenase Activity Assay as an Index of Soil Biological Activity. Biol. Fertil. Soils.

[80] Buysschaert B., Byloos B., Leys N., van Houdt R., Boon N. (2016). Reevaluating Multicolor Flow Cytometry to Assess Microbial Viability. Appl. Microbiol. Biotechnol..

[81] Veal D.A., Deere D., Ferrari B., Piper J., Attfield P.V. (2000). Fluorescence Staining and Flow Cytometry for Monitoring Microbial Cells. J. Immunol. Methods.

[82] Cavallin J.E., Durhan E.J., Evans N., Jensen K.M., Kahl M.D., Kolpin D.W., Kolodziej E.P., Foreman W.T., LaLone C.A., Makynen E.A. (2014). Integrated Assessment of Runoff from Livestock Farming Operations: Analytical Chemistry, in vitro Bioassays, and in vivo Fish Exposures. Environ. Toxicol. Chem..

[83] Tavares R.S., Martins F.C., Oliveria P.J., Ramalho-Santos J., Peixoto F.P. (2009). Parebens in Male Infertility—Is There a Mitochondrial Connection?. Reprod. Toxicol..

[84] Brown B.L., LePrell R.V., Franklin R.B., Rivera M.C., Cabral F.M., Eaves H.L., Gardiakos V., Keegan K.P., King T.L. (2015). Metagenomic Analysis of Planktonic Microbial Consortia from a Non-Tidal Urban-Impacted Segment of James River. Stand. Genom. Sci..

[85] Woodhouse J.N., Kinsela A.S., Collins R.N., Bowling L.C., Honeyman G.L., Holliday J.K., Neilan B.A. (2016). Microbial Communities Reflect Temporal Changes in Cyanobacterial Composition in a Shallow Ephemeral Freshwater Lake. ISME J..

[86] Morgan R.P., Kline K.M., Churchill J.B. (2013). Estimating Reference Nutrient Criteria for Maryland Ecoregions. Environ. Monit. Assess..

[87] Goodbred S.L., Patino R., Torres L., Echols K.R., Jenkins J.A., Rosen M.R., Orsak E. (2015). Are Endocrine and Reproductive Biomarkers Altered in Contaminant-Exposed Wild Male Largemouth Bass (*Micropterus salmoides*) of Lake Mead, Nevada/Arizona, USA?. Gen. Comp. Endocrinol..

[88] Patiño R., Goodbred S.L., Draugelis-Dale R., Barry C.E., Foott J.S., Wainscott M.R., Gross T.S., Covay K.J. (2003). Morphometric and Histopathological Parameters of Gonadal Development in Adult Common Carp from Contaminated and Reference Sites in Lake Mead, Nevada. J. Aquat. Anim. Health.

[89] Wang Y., Hammes F., DeRoy K., Verstraete W., Boon N. (2010). Past, Present and Future Applications of Flow Cytometry in Aquatic Microbiology. Trends in Biotechnology.

[90] Gray G.W., Wilkinson S.G. (1965). The Effect of Ethylenediaminetetra-acetic Acid on the Cell Walls of Some Gram-Negative Bacteria. Microbiology.

[91] Walberg M., Steent H.B. (2001). Flow Cytometric Monitoring of Bacterial Susceptibility to Antibiotics. Methods Cell Biol..

[92] Duquenoy A., Bellais S., Gasc C., Schwintner C., Dore J., Thomas V. (2020). Assessment of Gram-and Viability-Staining Methods for Quantifying Bacterial Community Dynamics Using Flow Cytometry. Front. Microbiol..

[93] Hammes F., Egli T. (2010). Cytometric Methods for Measuring Bacteria in Water: Advantages, Pitfalls and Applications. Anal. Bioanal. Chem..

